# The Hospital Magazines and Journals

**Published:** 1920-07-17

**Authors:** 


### The Hospital Magazines and Journals.

St. Thomas's Hospital Gazette.—A paper by Mr. J. E. Adams, which deals chiefly with the Lady Almoner's Department, retails a story by the late Sir Lauder Brunton of an old lady who kept a refreshment stall close to St. Bartholomew's Hospital, where she sold, amongst other delicacies, tarts flavoured with the hospital linctus. Anyone with a cough could get a bottle at the hospital, and, if they preferred cash to cough mixture, this good woman was always ready to buy for the embellishment of her famous medicated tartlets.

St. Bartholomew's Hospital Gazette.—A simple tablet has been set up in the chapel of St. Bartholomew's Hospital, inscribed with the words: "To the Memory of Arthur Watkins, Steward of this Hospital, who died on duty, 2nd May, 1919, aged 62 years, this Tablet is erected by the Governors of St. Bartholomew's Hospital, in Recognition of his unwearied zeal and devotion during 40 years' service."

Guy's Hospital Gazette.—The Editor refers to the enormous number of patients with symptoms and no physical signs who, for want of adequate stimulation, hamper the work of the special departments. It is proposed that a special department shall be established in every general hospital, to which shall be referred these "chronics" by the physician or surgeon, who after examination can find no evidence of organic disease, and can then transfer the patient to a department where simple treatment by suggestion or crude electrical apparatus will be available. An important point is that the physician-in-charge of the new department would accumulate material illustrating how often patients with symptoms and no signs develop definite organic disease while under treatment. It seems possible that a careful study of those patients with so-called functional disorders would result in our obtaining valuable information about the symptomatology of disease in its very earliest stages.

St. Mary's Hospital Gazette.—In referring to the American grant to University College Hospital Medical School, the Editor points out that the main significance of the grant is its hint to England that an up-to-date nation must spend money on the education of its doctors before it embarks on sweeping health schemes.

